# Chemo-and regioselective aqueous phase, co-acid free nitration of aromatics using traditional and nontraditional activation methods

**DOI:** 10.3389/fchem.2024.1400445

**Published:** 2024-05-15

**Authors:** Kelsey M. Plasse, Tara R. Mooney, Maxim Mastyugin, Maximilian Costa, Béla Török

**Affiliations:** Department of Chemistry, University of Massachusetts Boston, Boston, MA, United States

**Keywords:** nitration, aromatic electrophilic substitution, nitroaromatics, benzene derivatives, green synthesis, ultrasound, microwave, high hydrostatic pressure

## Abstract

Electrophilic aromatic nitrations are used for the preparation of a variety of synthetic products including dyes, agrochemicals, high energy materials, fine chemicals and pharmaceuticals. Traditional nitration methods use highly acidic and corrosive mixed acid systems which present a number of drawbacks. Aside from being hazardous and waste-producing, these methods also often result in poor yields, mostly due to low regioselectivity, and limited functional group tolerance. As a consequence, there is a need for effective and environmentally benign methods for electrophilic aromatic nitrations. In this work, the major aim was to develop reaction protocols that are more environmentally benign while also considering safety issues. The reactions were carried out in dilute aqueous nitric acid, and a broad range of experimental variables, such as acid concentration, temperature, time, and activation method, were investigated. Mesitylene and m-xylene were used as test substrates for the optimization. While the optimized reactions generally occurred at room temperature without any activation under additional solvent-free conditions, slight adjustments in acid concentration, stoichiometric equivalents, and volume were necessary for certain substrates, in addition to the activation. The substrate scope of the process was also investigated using both activated and deactivated aromatics. The concentration of the acid was lowered when possible to improve upon the safety of the process and avoid over-nitration. With some substrates we compared traditional and nontraditional activation methods such as ultrasonic irradiation, microwave and high pressure, respectively, to achieve satisfactory yields and improve upon the greenness of the reaction while maintaining short reaction times.

## 1 Introduction

The design of sustainable synthetic methods is more important today than ever (Török and Dranfield, 2018). The volume of the industrial production of chemicals, pharmaceuticals, agrochemicals, or consumer goods directly related to the chemical industry (e.g., plastics, dyes, etc.), makes the traditional ways impossible to continue due to the increasing environmental and safety concerns. This is particularly true for chemical processes that are carried out on a multibillion ton scale worldwide; where even a 10% cut of hazardous waste means a tremendous decrease in the total amount of waste to treat.

The nitration of aromatic compounds is one of these processes (Olah et al., 1989; Olah and Molnár, 2003). According to earlier data, 108 tons of nitro-aromatics are produced annually worldwide (Singh et al., 2015), nitrobenzene being the largest segment of these products with its global market size in 2022 of USD 9.76 billion and a predicted rise to 13 billion in 2027 (Nitrobenzene Global Market Report, 2023). Nitro-aromatics are used in large quantities in the pharmaceutical, high-energy materials/explosives, agrochemical, dye and perfume industries. In addition to their direct applications they serve as gateway chemicals to amines, hydroxylamines, aldehydes, carboxylic acids, among many possibilities, and through these precursors they are the indirect starting materials to heterocycles as well (Patra et al., 2022). As a prominent example, acetaminophen or paracetamol, the API in the drug Tylenol, is produced via the reduction and subsequent acylation of *p*-nitrophenol (Wilbert and De Angelis, 1961; Friderichs et al., 2007).

**FIGURE 1:**
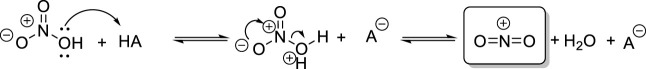
Generation of the nitronium ion from nitric acid with a strong co-acid.

The traditional nitration protocol involves the use of fuming nitric acid and another even stronger co-acid that can protonate nitric acid and generate the nitronium ion (NO_2_^+^), which is the commonly accepted reactive species in electrophilic aromatic nitrations (Figure 1), as originally elucidated by Ingold and Hughes (Esteves et al., 2003).

Due to the current, ever strengthening environmental regulations and safety standards, the traditional nitration protocol is not compatible with the sustainable and green production of chemicals. First of all, it is inherently hazardous, and the acid mixture is extremely corrosive. The negative environmental impact include: (i) the use of non-regenerable mineral acids, (ii) a significant amount of hazardous and corrosive acid waste, (iii) the disposal of the spent acid generates nitrous oxide (NO_x_) that are hazardous to the employees and also detrimental to the atmosphere (Song et al., 2019), (iv) often poor yield and functional group tolerance, coupled with low chemo- and regioselectivity, and (v) over-nitration which yields undesirable byproducts and results in more waste (Kroschwitz, 2004).

Due to the importance of nitration in many segments of the chemical industry the development of greener approaches is highly desirable and the extensive research progress has been frequently reviewed (Török et al., 2021a; Plasse and Török, 2024). Contemporary approaches focus on reducing waste (acid and undesirable byproducts) by increasing the yields, chemo- and regioselectivity of the reactions, using catalytic methods (Patel et al., 2021; Kokel et al., 2019), alternatives to aromatic electrophilic substitution such as C-H activation (Song et al., 2019), or radical chemistry (Khan et al., 2017; Gao et al., 2019; Song et al., 2020). Alternative activation methods such as sonication, microwave irradiation, or mechanochemistry have also been applied (Nandurkar et al., 2007; Török and Schäfer, 2021; Qian et al., 2021). Last but not least, alternative sources for the active species have been considered as well (Kuhn and Olah, 1961; Olah et al., 1982), including nitrite salts (Zhao et al., 2016), t-butyl nitrite (Dahiya et al., 2019), nitrates (Yan and Yang, 2013), and nitrous gases (Mori et al., 1995).

Continuing our recent efforts on the development of catalyst- and metal-free synthetic methods towards the development of practical syntheses of fine chemicals and biologically active compounds of interest (Cho et al., 2014; Pandey and Török, 2017; Kokel and Török, 2017; Xie et al., 2023; Vlocskó et al., 2024), in this work we describe the additional-catalyst and solvent-free nitration of aromatics using only dilute aqueous nitric acid as both nitronium ion source and self-catalyst. These methods eliminate the catalyst related recycling/regeneration and disposal steps and thus prevent considerable amount of toxic waste formation. The advantages of our method are the simple protocol, high atom economy producing water, a non-toxic compound, as the only by-product, and the energy efficient approach including room temperature reactions, or the application of non-traditional activation methods under safe conditions.

## 2 Materials and methods

### 2.1 General

Materials: All starting materials and solvents were commercially available and were purchased from Sigma-Aldrich or Alfa-Aesar and used without further purification. The fuming and 15.8 M nitric acid (HNO_3_) was purchased from ThermoFisher Scientitic.

A VWR Ultrasonic Cleaner, model 97,043–960, with an operating frequency of 35 kHz and an RF power of 48W was used. The bath was filled with DI water and used for optimizations and substrate scope.

The CEM Discover Focused Microwave synthesis system was used for deactivated substrates that did not have sufficient yields under conventional stirring and ultrasonication methods. An open system and stirring was used under microwave conditions by using a round bottom flask, stir bar, and condenser.

Reactions under High Hydrostatic Pressure were carried out in a Barocycler 2320EXT (Pressure BioSciences Inc.) instrument at room temperature and 3.8 kbar pressure in 150 µL high-pressure teflon reaction tubes sealed by teflon PCT MicroCaps.

### 2.2 Analytical methods

The ^1^H and ^13^C NMR analysis was carried out on a 400 MHz Agilent MM2 NMR spectrometer, in CDCl_3_ with either using the signal of tetramethylsilane or the residual solvent signal as reference. The temperature was 25°C (accuracy ±1°C). The mass spectrometric identification and purity determination of the products have been carried out by an Agilent 6,850 gas chromatograph-5973 mass spectrometer system (70 eV electron impact ionization) using a 30m long DB-5 type column (J&W Scientific). The HRMS data were obtained using an Agilent 7250 GC-QTOF mass spectrometer operated in electron impact ionization (EI, 70 eV) mode. The products were identified based on their retention times by comparing them to that of authentic samples (Lopez-Avila et al., 1991).

### 2.3 Catalyst-free nitration procedure

To a 10 or 25 mL round-bottomed flask with a stir bar, 8 mmol (or the desired amount) of starting material was added. A non-jacketed condenser was fitted on top (with a screw cap and o-ring). Nitric acid was added via syringe by slightly lifting the condenser, emptying the syringe into the flask, and re-fitting the condenser. The reaction was stirred for the appropriate amount of time. After the reaction time was over, the reaction was quenched with 10–15 mL of cold DI-water. The reaction mixture was then transferred to a separatory funnel. The reaction flask was rinsed with small amounts of ethyl acetate and transferred to the separatory funnel. About 20 mL of ethyl acetate was added to the separatory funnel. The organic layer was washed three times with 10% bicarbonate solution. The pH was checked after the second separation and third separations for neutral pH. If neutral pH was obtained, the organic layer was washed three times with brine. The organic layer was separated to an Erlenmeyer flask where it was dried with magnesium sulfate. The organic layer was transferred to a new 25 mL round-bottomed flask by gravity filtration of the magnesium sulfate. A GCMS analysis of the organic layer was carried out to determine product composition. Eventually, the washing of the product with sodium bicarbonate and drying with magnesium sulfate was replaced with a sodium carbonate column to both neutralize and dry the product at the same time as to cut down on the liquid waste of the process. Sodium carbonate (∼15 g) was placed into a glass frit and filtered through without the use of vacuum. However, sodium bicarbonate was still used to neutralize the aqueous layer before disposal.

## 3 Results

**FIGURE 2:**
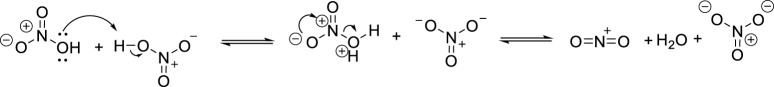
Self-protonation of HNO_3_ yielding NO2+ cation.

Our major goal in this work is to develop a green nitration protocol for a broad variety of aromatics that conforms to the major principles of Green Chemistry. Given the recent emergence of studies that reevaluate reactions without the use of catalysts (Brahmachari, 2017) of additional solvents it inspired us to attempt to carry out the nitration without additional co-acid catalyst to determine whether the *in situ* auto-ionization of nitric acid (Figure 2) is a sufficient source for the nitronium ion formation and the effective production of nitro-aromatics.

We have chosen mesitylene and m-xylene as aromatics to explore the optimization of the procedure. Our studies began by using fuming nitric acid and later gradually decreasing its concentration to determine the lowest acid concentration that is still able to accomplish the nitration reactions. In the first set of experiments we applied fuming HNO_3_ to explore the reaction conditions in the nitration of m-xylene.

The data described in Table 1 clearly shows that the reaction occurs effectively with fuming HNO_3_, and that the hydrocarbon/acid ratio significantly affects the product yield and distribution. A notable observation is that the presence of a catalyst or co-acid, in the form of K-10 montmorillonite, a well-known solid acid (Dasgupta and Török, 2008; Török et al., 2021b), significantly activates the system and over-nitration occurs in a considerable extent. If high acid ratio and K-10 are combined, nearly 60% of the product is an over-nitrated compound (Table 1, entry 4). It was also observed that the additional activating effect of the catalyst is negligible at high acid ratio; the over-nitration and general product distribution are nearly the same without K-10 (Table 1, entries 15, 16). We have also investigated the effect of the reaction time using 18.01 mmol HNO_3_. The data shows that although the abundance of isomer B is mainly the same and did not change over time, longer times, after 30 min, resulted in the formation of a sizeable amount of over-nitrated products (Table 1, entries 7–12). Interestingly, the reaction appears to reach an equilibrium-like state and after 45 min, the distribution of the different products remains approximately the same, even when the reaction time was doubled to 90 min (Table 1, entries, 9 and 12). These observations confirm the above mentioned drawbacks of traditional nitration. However, it appears that the reaction occurs with much higher selectivity when low acid ratio is applied in combination with short reaction times (Table 1, entries five and 7) indicating the feasibility of our goal.

**TABLE 1:** Effects of reaction conditions on the nitration of m-xylene using fuming nitric acida.

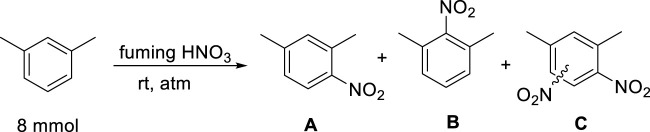						
Entry	fuming HNO_3_ (mmol)^b^	Additive/catalyst	time (min)	A (%)^c^	B (%)^c^	C (%)^c^
1	12.01	K-10 (0.5 g)	103	75	9	16
2	18.01	K-10 (0.5 g)	100	74	9	13
3	24.01	K-10 (0.5 g)	90	64	12	24
4	48.02	K-10 (0.5 g)	90	36	6	58
5	12.01	-	15	92	8	0
6	12.01	-	90	85	12	3
7	18.01	-	15	94	6	0
8	18.01		30	95	5	0
9	18.01		45	85	7	9
10	18.01		60	81	8	8
11	18.01		75	84	9	7
12	18.01	-	90	85	8	7
13	24.01	-	15	87	13	0
14	24.01	-	90	91	9	0
15	48.02	-	15	37	5	58
16	48.02	-	90	39	5	56

^a^ General reaction conditions: fuming nitric acid, m-xylene, ambient temperatute, conventional stirring.

^b^ Calculated based on literature fuming HNO_3_ density of 1.513 g/mL (Daştan et al., 2012; Kokel et al., 2017).

^c^ Determined by GCMS.

As a next step it was decided to investigate the effect of the nitric acid concentration on the yield and selectivity of the reaction. Based on a density of about 1.5 g/mL, fuming nitric acid has a concentration of 24.01 M. As our goal is to achieve the nitration by using dilute HNO_3_, we created a dilution series to investigate the effect of HNO_3_ concentration on the outcome of the reaction. The starting concentration was 15.8 M, as this nitric acid concentration is commercially widely available. The data are tabulated in Table 2.

**TABLE 2:** Effect of aqueous nitric acid concentration on the nitration of m-xylene.

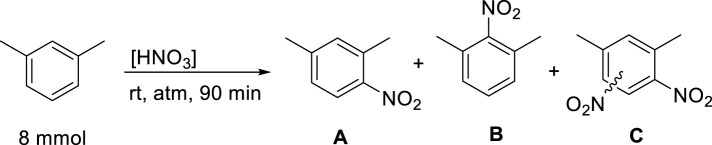						
Entry		[HNO_3_] (M)	Total conversion (%)^a^	A (%)	B (%)	C
1		15.8	66	85	15	0
2		14	23	80	20	0
3		12	1	90	10	0
4		10	0	0	0	0
5		8	0	0	0	0

^a^ Determined by GCMS.

The most important observation during the above dilution experiments was that none of the concentrations yielded di-nitro products. This is an important step forward as the major goal is to achieve selective mono-nitration. It appears that the commercially available 15.8 M sample provided reasonable yield and 4-nitro regioselectivity. Further diluting this sample to 14 M resulted in a considerable drop in conversion, while the selectivity remained similar. The 12 M nitric acid appears to be the lowest concentration where product formation was observed, however, the conversion is nearly negligible at 1%. Although the selectivity improved to 90/10 for the 4-nitro product, the low conversion makes the use of this concentration impractical.

To further explore the effect of reaction conditions, we have continued testing the reactions using the commercial 15.8 M sample as a nitrating agent. At this time we wanted to focus on the effect of different conditions on the yield of the reaction, thus we selected mesitylene as a substrate as this compound only yields one mono-nitro product due to the high level of symmetry in the compound. The results are summarized in Table 3.

**TABLE 3:** Effect of reaction conditions on the nitration of mesitylene.^a^

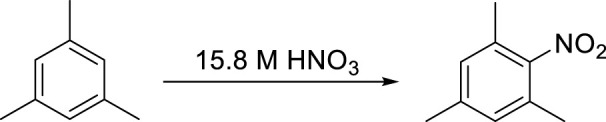							
Entry	HNO_3_ (mL)		Mesitylene (mL)	molar ratio HNO_3_/Mes	Conditions	Time (min)	Conversion (%)^b^
1	5.0		0.5	22	ultrasound, rt	30	34
2	5.0		0.5	22	ultrasound, rt	40	91
3	5.0		0.5	22	ultrasound, rt	50	94
4	5.0		0.5	22	ultrasound, rt	90	100
5	5.0		0.5	22	conventional stirring, rt	60	97
6	6.0		0.5	26.3	ultrasound, rt	60	96
7	6.0		0.5	26.3	conventional stirring, rt	60	97
8	3.5		0.5	15.4	ultrasound, rt	60	95
9	3.5		0.5	15.4	conventional stirring, rt	60	90
10	2.5		0.5	11.0	ultrasound, rt	60	100
11	2.5		0.5	11.0	conventional stirring, rt	60	100
12	2.0		0.5	8.8	ultrasound, rt	60	39
13	2.0		0.5	8.8	conventional stirring, rt	60	88
14	1.5		0.5	6.6	ultrasound, rt	60	2
15	1.5		0.5	6.6	conventional stirring, rt	60	26
16	1.0		0.5	4.4	ultrasounds, rt	60	3
17	1.0		0.5	4.4	conventional stirring, rt	60	3
18	0.5		0.5	2.2	ultrasound, rt	60	1
19	0.5		0.5	2.2	conventional stirring, rt	60	4

^a^ General reaction conditions: 15.8 M nitric acid, mesitylene, ambient temperatute, mixing.

^b^ Determined by GC-MS.

The data shown in Table 3 describe the results of experiments with different amounts of acid and hydrocarbon, changing molar ratios, as well as reactions carried out with conventional stirring and ultrasound, respectively. The rationale regarding the use of ultrasound was that in the nitration due to the presence of the aqueous HNO_3_ and the non-polar hydrocarbon, one is dealing with a two-phase system and the reaction rates are likely limited by the mass transfer over the phase boundary. In addition to its efficacy as an activation method (Török et al., 2001; Török et al., 2002), sonication is also well-know for its mixing efficiency and its strong contribution to green synthesis (Mishra and Török, 2021; Mishra and Török, 2023). The data in Table 3, however, indicates that although sonication works well under most conditions, producing nearly quantitative yields, it does not seem to enhance the results as compared to the conventional stirring of the reaction mixture (Table 3, entries 3 vs. 5). Another important observation made in this series of experiments, is that decreasing the amount of HNO_3_, therefore the HNO_3_/hydrocarbon ratio, will negatively affect the yields. In the range of 6–3.5 mL of HNO_3_ to 0.5 mL mesitylene (molar ratios are 26.3 to 15.4) the reaction appears to be unaffected by the decreasing amount of nitric acid, the yields remain stable in the 90%–95% range (Table 3, entries 5–11). Further decreasing the nitric acid volume, while all other conditions are unchanged, results in a gradual drop in the yields. Although 2.5 mL HNO_3_ still yields 100% by sonication and also by stirring, decreasing the HNO_3_ amount to 2 mL or even lower results in a sudden drop in product yield to 39% by sonication, and a more gradual decrease to the same level by stirring (Table 3, entries 10,11 vs. 12,13). Thus, the molar ratio of the nitric acid to the aromatic hydrocarbon is a determining factor in the outcome of the reaction. As expected, the nitration occurs effectively when the NO_2_^+^ source is in considerable excess, and when the molar ratios get closer to four or two the nitration will not occur with acceptable yields (Table 3, entries 16–19). A very promising feature of the reaction conditions is that the nitration occurs at ambient temperature, thus, no formal energy investment is required to initiate the nitration. Interestingly, the addition of extra amount of water to the reaction mixture at different molar ratios, i.e., effectively decreasing the concentration of the acid in the mixture without lowering the molar ratio, resulted in significant drop in the yields, even at the highest molar ratio. When additional water was introduced to the system to carry out the nitration with more dilute nitric acid (data not shown), the yields were mostly in the single digits, but never higher than 29%. This indicates that the reaction is driven by multiple factors; in this case, both the molar ratio and the actual HNO_3_ concentration represent key factors. When these numbers are below a certain limit the reaction ceases to occur.

In the above experiments, the following main conclusions led us to optimized conditions that will be used in determining the scope of the reaction. (i) Conventional stirring appears to be as effective as the sonication, thus sonication does not need to be used. (ii) The decrease in the HNO_3_/hydrocarbon ratio will result in diminishing yields below the 8.8 M ratio (Table 3, entries 14–19). Further decreasing ratios to four or two will only yield product formation in single digit percentiles (Table 3, entries 16–19). (iii) Despite all the shortcomings, the aqueous 15.8 M HNO_3_ resulted in effective nitration essentially indicating that aromatic electrophilic nitration of hydrocarbons can be carried out without the use of fuming nitric acid, or any other strong co–acid or other catalyst. This simple modification, while it does not decrease the efficacy, can improve selectivities, while considerably decreasing the environmental impact of the protocol.

Based on the above findings it was decided to explore the scope of the reaction. Several aromatics have been selected for these reactions bearing a broad range of substituents, including electron donating and withdrawing groups. In all cases the above identified optimized conditions were applied. However when necessary, a limited additional optimization was carried out to achieve the highest possible yield for the reactions. Conventional stirring, sonication, microwave irradiation and high pressure were considered as reaction conditions, and the best result obtained is shown for each substrate. The results are summarized in Table 4.

**TABLE 4:** Nitration of aromatics with co-acid-free aqueous nitric acid.^a^

Entry	Substrate (S)	molar ratio HNO_3_/S	Conditions (min)	Product	Conversion (%)^b^
1	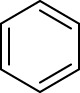	11	stirring, rt, 2.6 mL HNO_3_/0.4 mL benzene, 90	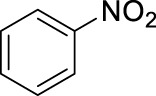	66
2	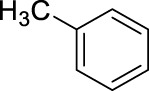	11	ultrasound, rt, 2.6 mL HNO_3_/0.4 mL toluene, 120	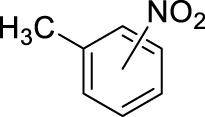	100 (o/m/p: 53/4/43)
3	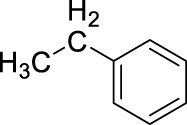	11	ultrasound, rt, 2.6 mL HNO_3_/0.4 mL toluene, 120	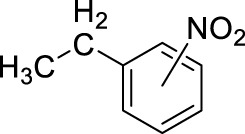	74 (o/m/p: 39/7/54)
4	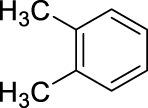	10	stirring, rt, 2.6 mL HNO_3_/0.45 mL o-xylene, 90	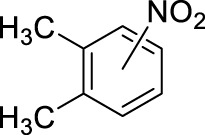	60 (3/4-NO2: 31/69)
5	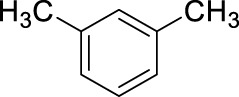	11	stirring, rt, 2.6 mL HNO_3_/0.45 mL m-xylene, 90	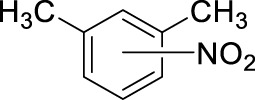	88 (2/4-NO2: 23/77)
6	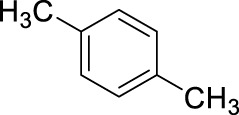	11	ultrasound, rt, 2.6 mL HNO_3_/0.45 mL p-xylene, 120	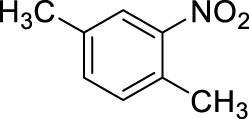	71
7	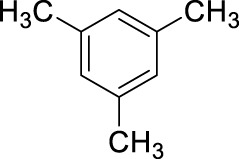	11	stirring, rt, 2.5 mL HNO_3_/0.5 mL mesitylene, 60	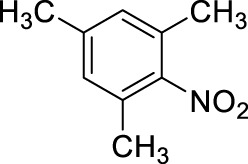	100
8	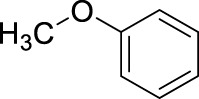	11	ultrasound, rt, 2.6 mL HNO_3_/0.4 mL anisole, 120	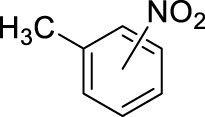	95 (o/m/p: 19/0/81)
9	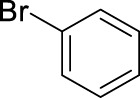	11	microwave, 50°C, 2.6 mL HNO_3_/0.4 mL bromobenzene, 30	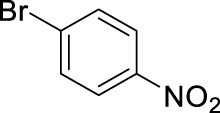	93 (o/m/p: 2/0/98)
10	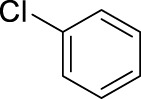	11	microwave, 85°C, 2.6 mL HNO_3_0.4 mL chlorobenzene, 30	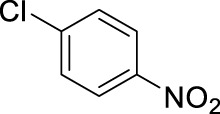	92 (o/m/p: 34/0/58)
11	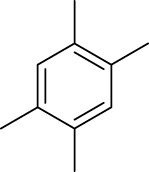	11	ultrasound, rt, 2 mL HNO_3_/0.3858 g 1,2,4,5-tetramethyl-benzene, 120	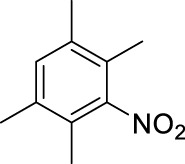	42

^a^ General reaction conditions: 15.8 M nitric acid, substrate in varying ratios, ambient temperature or 50°C, conventional stirring/ultrasounds or microwaves.

^b^ Determined by GC-MS.

The data shown in Table 4 indicate that the aqueous (15.8 M) HNO_3_ can achieve the effective nitration of a broad variety of compounds including hydrocarbons and other substituted benzene derivatives. The analysis of the data is provided below.

**FIGURE 3:**
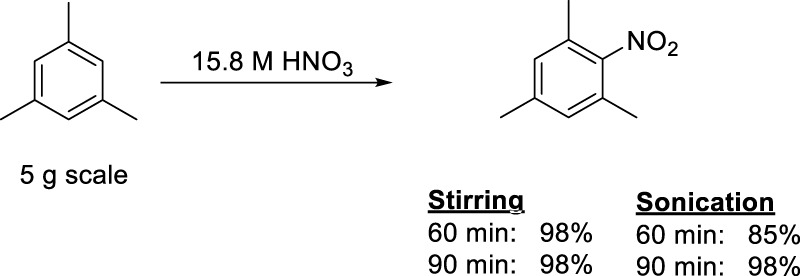
Nitration of mesitylene at a preparative (5 g) scale by conventional stirring and sonication.

After the success of the above described small scale experiments, it was decided to investigate the potential scale up possibilities of our nitration protocol. The scale of the experiments were increased by a factor 10 to an approximately 5 g scale for the product. At this level of the investigations, we simply multiplied the amount of substrate (mesitylene) and the HNO_3_ used by ten. Despite this simple scale-up protocol, the data, shown in Figure 3, clearly indicated that the preparative scale reactions occurred with the same high yields (up to quantitative yields) even after only 60 min reaction time with stirring. The sonicated samples required slightly more time (90 min) to reach the same yield. No byproduct (i.e., di-nitro derivative) formation was observed in either set of experiments. These observations suggest that the co-acid-free protocol is scalable without significant alteration of the original method.

## 4 Discussion

The data, including the optimization experiments (Tables 1–3) and investigation of the scope (Table 4) clearly proved that our hypothesis, namely, that the aqueous diluted HNO_3_ can be effectively used for nitration reactions of aromatics, was correct. During the optimization we have concluded several points, as discussed above that were used during the studies of the scope. First of all, as a benchmark, benzene readily underwent nitration producing nitrobenzene in a moderate yield (66%, Table 4, entry 1), in a relatively short reaction. Considering the significant boiling point difference between the two compounds, benzene can easily be separated from the product and recycled. Most importantly, no byproduct formation was observed, thus the reaction occurred with 100% selectivity, which eliminates a tedious and energy demanding/waste producing separation step. As a general observation; under the reaction conditions applied, even when using microwave irradiation, the formation of di-nitro products were never observed, which is a major step forward.

Table 4 also reveals that the typical differences between the two main groups of aromatic substituents, namely, activating vs. deactivating, are on full display in these nitrations. Considering that reactivity and selectivity are commonly in a reverse relationship, it is well demonstrated that most compounds with electron donating groups, such as methyl, ethyl, or multiple methyl groups, yielded the nitration products in good to excellent yields, up to quantitative yield in case of mesitylene, and toluene (Table 4 entries two and 7). In addition, anisole also underwent mono-nitration in 95% yield (Table 4, entry 8). While the o/p selectivity is somewhat moderate in the case of toluene, it is quite high (80% para) for anisole. Obviously, the regioselectivity is the result of complex features, not only the electronic effect. As a secondary force, the steric effects also contribute to the regioselectivity, while there are more ortho-product formed in the nitration of toluene, this selectivity was reversed when ethyl benzene, with a larger substituent, was used as a substrate (Table 4, entries two and 3). It is also shown that deactivated benzene derivatives, such as bromobenzene and chlorobenzene did not undergo nitration under the typical conditions. However, when microwave irradiation (Nitric acid and fuming, 2021) was used at a moderate temperature (50°C) excellent product yield (93% yield and 98% para-selectivity) was obtained for bromobenzene (Table 4, entry 9), and some product formation (18% yield) was detected with chlorobenzene et the same temperature. The decreased yield for chlorobenzene is attributed to the higher electron negativity and thus stronger deactivating effect of chlorine compared to that of bromine. This lower activity can be countered by elevating the reaction temperature to 85°C under microwave conditions, which yielded 92% product (Table 4, entry 10). The data indicates that although microwave activation appears to enable the nitration of deactivated aromatics with aqueous HNO_3_ (Table 4, entries 9, 10), as well, more studies are needed to establish optimum conditions for these reactions. Many other deactivated benzene derivatives were investigated (e.g., acetophenones, benzoic acids, etc.), however, under the current conditions (rt, aq. HNO_3_) these compounds were not reactive.

As an interesting fact, it was observed in the optimization experiments that sonication, as a form of activation and also enhanced mixing, did not result in notable benefits in those reactions; the yields were the same or somewhat lower that those obtained with conventional stirring. Despite, in testing the scope, several substrates gave higher yields with sonication, and thus reported in Table 4. For example, toluene (100% yield, vs. 79% with stirring, Table 4 entry 2), ethylbenzene (74% yield, vs. 54% with stirring, Table 4 entry 3), p-xylene (71% yield, vs. 30% with stirring, Table 4 entry 6) all gave considerably better yields with ultrasonic irradiation. Even bromobenzene gave 5% yield upon sonication vs. no product under stirring. Although the data does not allow widespread generalization, it certainly highlights that every substrate should be treated as an individual case in terms of optimization for the mixing and activation.

It is also worth noting that another non-traditional activation method, high hydrostatic pressure (HHP), was also used as an attempt to improve the yields. Interestingly, the exact opposite was found; HHP resulted in a major drop in reaction yields when nitrating ethylbenzene (10% HHP yield vs. 74% stirring), m-xylene (16% HHP vs. 88% stirring), p-xylene (10% HHP vs. 71% sonication) or mesitylene (16% HHP vs. 100% stirring). As the HHP effect is proposed to be based on the activation volume (ΔV‡) and reaction volume (ΔV) of processes, the data suggests that nitration has positive ΔV‡ and ΔV values, in which case the pressure works against the reaction (Xie et al., 2023, Xie and Török, 2025^1^), as observed here.

## 5 Conclusion

Based on the above reported data, one can conclude that aqueous dilute nitric acid without the application of any co-acid or other catalyst, can be applied for the selective mono-nitration of many aromatics, providing moderate to excellent yields and often high selectivities. These conditions completely avoid the double nitration of the substrates, which is a significant advantage when compared to fuming nitric acid driven reactions, or other conditions when a strong co-acid is used. Other benefits are the use of a commercially available diluted nitric acid (15.8 M), and the clear safety benefits as opposed to those with the use of strong mineral co-acids, as well as the significantly lower amount of waste produced. Our results indicate that an investigation of the scale up of these nitrations using diluted aq. Nitric acid to industrially relevant scales warrant considerations.

## Data Availability

The original contributions presented in the study are included in the article/Supplementary material, further inquiries can be directed to the corresponding author.

## Footnotes

1 Xie, G., and Török, B. (2025). “Chemical synthesis with high hydrostatic pressure,” in Encyclopedia of green chemistry (China: Elsevier). (accepted)
